# Rapid-Hardening Engineered Cementitious Composites with OPC–SAC Binder and Recycled Fine Aggregates: Mechanical Properties and Micro-Mechanisms

**DOI:** 10.3390/ma19112355

**Published:** 2026-06-02

**Authors:** Zhejun Su, Jingwei Gong, Xinjie Wang, Minqi Hua

**Affiliations:** 1 College of Hydraulic and Civil Engineering, Xinjiang Agricultural University, Urumqi 830052, China; m15755029138@163.com; 2 Xinjiang Key Laboratory of Hydraulic Engineering Security and Water Disasters Prevention, Urumqi 830052, China; 3 School of Urban Construction, Changzhou University, Changzhou 213164, China; 4 School of Civil Engineering & Architecture, Wuhan University of Technology, Wuhan 430070, China

**Keywords:** engineered cementitious composites, sulfoaluminate cement, recycled fine aggregate, strain-hardening, early-age strength, multiple-cracking behavior

## Abstract

To address the slow early-age strength development of conventional engineered cementitious composites (ECCs), which limits their applicability in rapid-hardening engineering, and to promote the efficient resource utilization of construction and demolition waste, this study proposes a recycled high-ductility early-strength ECC (RHE-ECC) prepared using an ordinary Portland cement (OPC)–sulfoaluminate cement (SAC) composite binder, with recycled fine aggregate (RFA) fully replacing natural fine aggregate (NFA) and PVA fibers incorporated. The effects of the SAC replacement level and water–binder ratio (W/B) on the workability and mechanical properties of RHE-ECC were systematically investigated. The mechanical performance differences between RFA and NFA systems under the SAC–OPC composite binder were compared, and the micro-mechanisms by which RFA regulates the multiple-cracking behavior of ECC were elucidated through XRD and SEM analyses. The results indicate that at a SAC replacement level of 25%, the RHE-ECC achieves a 1 d compressive strength of 19.3 MPa while maintaining a 28 d compressive strength of 47.9 MPa, establishing a favorable balance between rapid early-age strength gain and sustained long-term development. At a W/B of 0.27, the RHE-ECC attains a 28 d ultimate tensile strain of 3.13%. This study systematically investigates, for the first time, the synergistic effects of the OPC-SAC composite cementitious system and full RFA replacement on the strain-hardening behavior of ECC, revealing that the porous old mortar layer of RFA weakens the ITZ, thereby reducing matrix fracture toughness and promoting multiple cracking, which enhances tensile strain capacity. These findings provide a theoretical foundation and technical support for the application of green, high-ductility cementitious composites in rapid-hardening engineering.

## 1. Introduction

Concrete is the most widely used construction material in modern building engineering. However, it is prone to cracking during service. The occurrence of cracks not only compromises the aesthetic appearance and functional performance of structures, but more critically, cracks serve as rapid transport channels for harmful agents, accelerating material deterioration and significantly reducing the service life of structures [1,2,3,4]. Meanwhile, global demand for sand and gravel aggregates continues to grow, while excessive extraction of natural sand and gravel resources has given rise to a series of environmental, social, and economic challenges across various regions worldwide. In this context, how to efficiently enhance the recycling and utilization of recycled aggregates to achieve resource conservation and environmental sustainability has become a critical issue that urgently needs to be addressed in the field of construction materials [5,6,7,8]. Engineered cementitious composites (ECCs), owing to their high ductility, exceptional crack control capability, and favorable durability, are regarded as ideal materials for structural strengthening and repair, which opens up new avenues for the application of recycled fine aggregates (RFAs) [9,10]. However, conventional ECC suffers from the drawback of slow early-age strength development, making it difficult to meet the demands of rapid-hardening engineering. This need has driven the development of early-strength ECC incorporating full recycled fine aggregates.

Sulphoaluminate cement (SAC), with anhydrous calcium sulfoaluminate (C_4_A_3_S^−^) as its principal clinker mineral, offers notable advantages, including rapid-hardening high early strength, low shrinkage, and low alkalinity, while its production process reduces CO_2_ emissions by 25–35% [11,12,13]. Leveraging these merits, researchers have incorporated SAC into the matrix design of ECC to overcome the slow early-age strength development and excessive drying shrinkage inherent in conventional OPC-based ECC. Previous studies have demonstrated that SAC-based ECC can achieve considerable mechanical strength within a few hours, accompanied by significantly reduced drying shrinkage, which is of substantial practical value for rapid-hardening engineering [14,15,16,17]. However, the relatively high production cost of SAC, combined with the predominance of thermodynamically unstable ettringite (AFt) among its hydration products, renders the system susceptible to strength regression at later ages [18,19]. No study has systematically investigated the combination of an OPC–SAC composite binder with recycled fine aggregates in ECC, leaving a critical knowledge gap regarding whether the rapid-hardening characteristics of SAC can be maintained when the matrix contains a high volume of porous RFA that competes for mixing water. The present study addresses this specific research gap. Consequently, how to harness the early-strength and shrinkage-reducing benefits of SAC while mitigating its long-term performance deficiencies has become a key issue in ECC material design [20,21]. In this context, blending OPC and SAC to establish an OPC–SAC hybrid cementitious system is expected to leverage the synergistic and complementary effects between the hydration products of the two cements [22,23,24]. Such an approach holds the promise of enhancing the long-term mechanical stability and durability of the system while retaining the advantages of rapid early-age strength development and low shrinkage, thereby offering a new technical pathway for the mix design optimization of high-performance ECC.

RFA, obtained through the crushing and sieving of waste concrete, currently has a utilization rate far lower than that of recycled coarse aggregate. Compared with natural sand, RFA—having undergone the in-service damage of the original concrete and subsequent mechanical crushing—typically carries a substantial amount of residual old mortar adhered to its particle surfaces, along with high internal porosity and well-developed micro-cracks [25,26,27,28]. These characteristics result in elevated water absorption, a high crushing index, and a weak interfacial transition zone (ITZ) between the RFA and the fresh cement paste [29,30,31,32]. The inherent defects of RFA alter the failure mode of the matrix to a certain extent, which is beneficial to the enhancement of the ductility of ECC [33,34]. Based on this mechanism, RFA has been successfully applied in conventional OPC-based ECC. However, systematic research on the incorporation of RFA into ECC with an SAC–OPC composite cementitious system remains lacking. This research direction holds the promise of achieving high-value utilization of construction and demolition waste while maintaining or even enhancing the early-age strength and tensile ductility of the material, thereby providing new insights for advancing the development of ECC toward greater sustainability and cost-effectiveness. Multiple cracking behavior is the defining constitutive characteristic that distinguishes ECC from conventional concrete, and is of significant importance for limiting catastrophic brittle failure, enhancing structural toughness, and improving energy dissipation capacity. The investigation of the multiple-cracking mechanism in this study aims to explain the regulatory effects of RFA on the constitutive behavior of the material from a micromechanical perspective, providing a theoretical basis for material design.

This study aims to address the technical bottlenecks of slow setting speed and insufficient early-age strength development in conventional ECC while advancing the application of RFA in rapid-hardening engineering. This study also aims to determine the optimal SAC content that balances rapid early-age strength development with long-term performance in the RFA-ECC system, optimize the water–binder ratio to achieve the best comprehensive tensile performance at the identified optimal SAC level, and elucidate the micro-mechanisms by which RFA regulates multiple-cracking behavior within the rapid-hardening composite binder system through comparative analysis of RFA and NFA systems combined with XRD and SEM characterization. The findings of this study are expected to provide a theoretical basis and technical support for the engineering application of RFA in early-strength ECC, and to offer new perspectives for the development of novel cementitious composites that integrate high performance with sustainability.

## 2. Experiment

### 2.1. Materials

**Figure 1:**
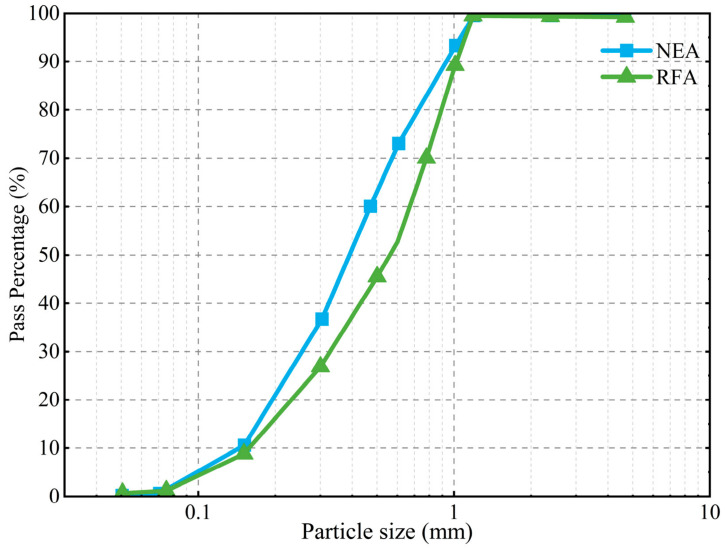
Particle size distribution of aggregates.

The cementitious materials used in this study comprised P·O 52.5 ordinary Portland cement (Conch Cement Plant) (Wuhu, China) R·SAC 42.5 rapid-hardening SAC (Tangshan Polar Bear Building Materials Co., Ltd.) (Tangshan, China), Class I fly ash (FA) (Changzhou Hutang Thermal Power Plant) (Changzhou, China), and highly reactive silica fume (SF) (Henan Yixiang New Building Materials Technology Plant) (Sanmenxia, China). Their chemical compositions are presented in Table 1. The mineral composition of R·SAC 42.5, comprised approximately 63% ye’limite (C_4_A_3_S^−^), 16% belite (C_2_S), 7% anhydrite (CS^−^), and 14% minor phases, including gehlenite and ferrite. The Class I fly ash had a median particle size (D50) of 12.5 μm and a specific surface area of 380 m^2^/kg (Blaine), and exhibited a characteristic spherical morphology. The silica fume had a BET specific surface area of 18,500 m^2^/kg and a median particle size of approximately 0.15 μm, and exhibited an amorphous spherical morphology. PVA fibers were incorporated as a matrix-toughening reinforcement, with their key performance parameters listed in Table 2. Natural fine aggregate (NFA) was locally sourced river sand, while RFA was produced by crushing and grinding locally collected waste concrete. The RFA was prepared from locally collected waste concrete through primary crushing by a jaw crusher, secondary crushing by a cone crusher, and screening to obtain the 0.075–1.18 mm fraction, then oven-dried at 105 ± 5 °C for 24 h to constant mass and stored in sealed containers. The particle size of both aggregates ranged from 0.075 to 1.18 mm. The particle size distribution curves of the two aggregates are shown in Figure 1, and their physical properties are summarized in Table 3. In the mix design, RFA was used in the oven-dry condition, and additional water (Table 4, sorbed water column) was calculated based on the measured 24-h water absorption (8.71%) to compensate for water absorbed by RFA during mixing, ensuring that the effective water (Effective Water column) corresponded to the designed W/B ratio. In addition, a polycarboxylate-based high-range water reducer (HRWR) with a water-reducing efficiency of 25% was added during the mixing process.

### 2.2. Mix Proportion

In this study, RFA was used as a complete replacement for NFA in the preparation of ECC, with the aim of enhancing cost-effectiveness and environmental sustainability. The experimental variables included substitution rate of SAC Cement, W/B, and curing age. Five SAC replacement levels were adopted—0%, 25%, 50%, 75%, and 100%—to cover the commonly investigated substitution range. Preliminary trial results indicated that even a relatively low SAC replacement ratio could yield a notable improvement in ECC performance while maintaining favorable economic viability, thereby demonstrating promising potential for the effective optimization of ECC. On this basis, a SAC replacement ratio of 25% was selected for further investigation, under which five W/B values—0.21, 0.24, 0.27, 0.30, and 0.33—were systematically examined to elucidate the evolution of mechanical properties of ECC at different curing ages (1 d, 3 d, 7 d, and 28 d). In all mix proportions, the FA content was fixed at 40% and the SF content at 10% of the total cementitious materials by mass. Furthermore, additional water was incorporated into each mix design to compensate for the water absorption of both RFA and NFA. The dosage of the high-range water reducer was uniformly controlled at 0.8% of the total binder mass to eliminate its ancillary effects on the properties of hardened concrete, while ensuring uniform dispersion of PVA fibers throughout the matrix [35]. Detailed mix proportions are provided in Table 4.

### 2.3. Test Methods

Although the powder components in the matrix are relatively fine and similar in particle size, which is conducive to uniform dispersion, the incorporation of a substantial volume of PVA fibers in ECC makes inadequate mixing prone to fiber agglomeration and non-uniform distribution. A well-designed mixing protocol is therefore critical to ensuring specimen quality.

**Figure 2:**
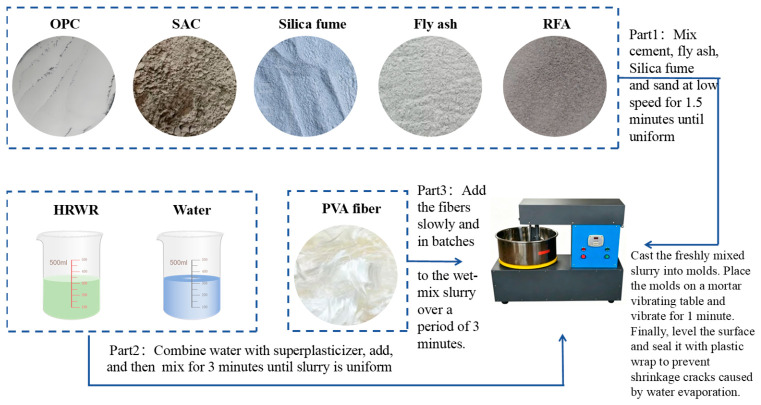
Test procedure.

All mixtures were prepared using a UJZ-15 planetary mortar mixer (Cangzhou Kexing Instrument Equipment Co., Ltd.) (Cangzhou, China) with a rated capacity of 15 L. The mixer features a counter-rotating planetary action: blades rotate clockwise at 80 ± 4 rpm while the bowl rotates counterclockwise at 60 ± 2 rpm. Each batch produced approximately 7–8 L of fresh ECC paste. In this study, a three-stage mixing procedure was adopted. Figure 2 presents a schematic diagram of the mixing procedure. In the first stage (dry mixing), cement, FA, SF, and fine aggregate were added to the mixer and dry-mixed for 1 min to achieve thorough homogenization of the powder components. In the second stage (wet mixing), the pre-blended mixture of water and high-range water reducer was slowly introduced into the dry mix, followed by continued mixing for 3 min until a homogeneous paste with adequate workability for subsequent fiber incorporation was obtained. The fluidity of the final mixture (after fiber addition) was measured to be approximately 120 mm, as reported in Section 3.1.1. In the third stage (fiber incorporation), PVA fibers were gradually added in small batches into the wet paste, with mixing continued for approximately 3 min until the fibers were visually confirmed to be uniformly dispersed throughout the matrix with no visible agglomeration.

After mixing, the fresh paste was cast into molds and placed on a vibrating table for 1 min to eliminate entrapped air and ensure compaction. The specimen surfaces were then leveled and covered with polyethylene film to prevent early-age shrinkage cracking caused by moisture evaporation. The specimens were demolded 24 h after casting and immediately transferred to a standard curing room maintained at a temperature of (20 ± 2) °C and a relative humidity of no less than 95%, where they were cured until the designated ages prior to testing. ECC specimens with dimensions of 40 mm × 40 mm × 160 mm were used for compressive and flexural strength tests. Dog-bone-shaped ECC specimens were employed for uniaxial tensile tests.

#### 2.3.1. Setting Time and Fluidity

The initial and final setting times of fresh paste were determined in accordance with GB/T 1346-2011 [36] on cementitious paste without fine aggregate or fibers (cement + FA + SF + water + HRWR). The fluidity test was conducted following GB/T 2419-2005 [37] on the complete ECC mixture including PVA fibers, prepared using the three-stage mixing procedure. The mixture was placed into a truncated cone mold, and after jolting on the flow table, the spread diameters were measured along two perpendicular directions and averaged.

#### 2.3.2. Compressive and Flexural Strength Tests

Flexural and compressive strengths were determined in accordance with GB/T 17671-2021 [38] using a computer-controlled constant-loading testing machine at loading rates of 50 N/s for flexural tests and 1.5 kN/s for compressive tests. Three prismatic specimens (40 mm × 40 mm × 160 mm) were prepared for each mix group. Three-point bending flexural tests were first performed, followed by compressive tests on the six resulting half-prism fragments. Compressive strength data were processed strictly in accordance with GB/T 17671–2021, taking the arithmetic mean of six measurements with outliers exceeding ±10% of the mean discarded or the set voided as specified, with results reported to 0.1 MPa.

#### 2.3.3. Uniaxial Tensile Test

To evaluate the tensile ductility of RHE-ECC, uniaxial tensile tests were conducted using dog-bone-shaped flat specimens in accordance with the recommendations of the Japan Society of Civil Engineers (JSCE) [39].

**Figure 3:**
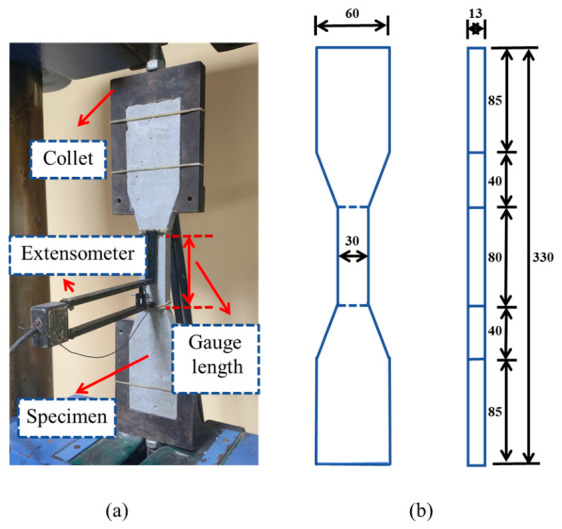
Dog-bone-shaped specimen for tensile test: (**a**) test instrument. and (**b**) geometric size (unit: mm).

Uniaxial tensile tests were conducted using dog-bone-shaped flat specimens (total length 330 mm, gauge section 30 mm × 13 mm) mounted on a WA-600C electro-hydraulic servo universal testing machine (Cangzhou Kexing Instrument Equipment Co., Ltd.) (Cangzhou, China) with wedge-type grips. A YSJ25-80 extensometer (Shanghai Leao Test Instrument Co., Ltd.) (Shanghai, China). (gauge length 80 mm) was installed at the middle section to measure tensile deformation, with loading applied at a constant displacement rate of 0.5 mm/min until fracture. A minimum of three specimens were tested per group and averaged (see Figure 3).

For each mix design at each curing age, three prismatic specimens (40 mm × 40 mm × 160 mm) were prepared for flexural testing, with the six resulting half-prism fragments used for compressive testing; a minimum of three dog-bone-shaped specimens were prepared for uniaxial tensile testing; fluidity and setting time tests were repeated three times per group.

## 3. Results and Discussion

### 3.1. Effect of SAC Cement Substitution Rates

#### 3.1.1. Fluidity and Setting Time

**Figure 4:**
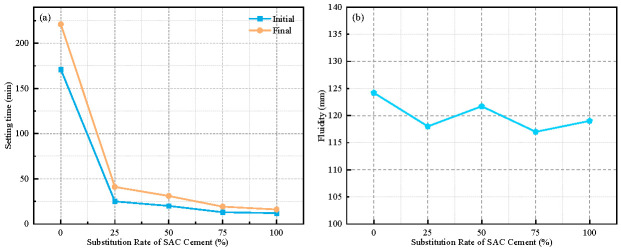
Workability under various substitution rates of SAC cement: (**a**) setting time; (**b**) flowability.

Figure 4 shows the fluidity and setting time under different SAC cement replacement levels. As seen in Figure 4a, when the replacement ratio varies from 0% to 100%, the fluidity remains around 120 mm, indicating that the SAC cement replacement level has little influence on the fluidity. Figure 4b demonstrates that the SAC cement content has a significant effect on the setting time. At a 25% replacement ratio, the initial setting time is 25 min. As the replacement ratio increases, the initial setting time gradually shortens.

**Figure 14:**
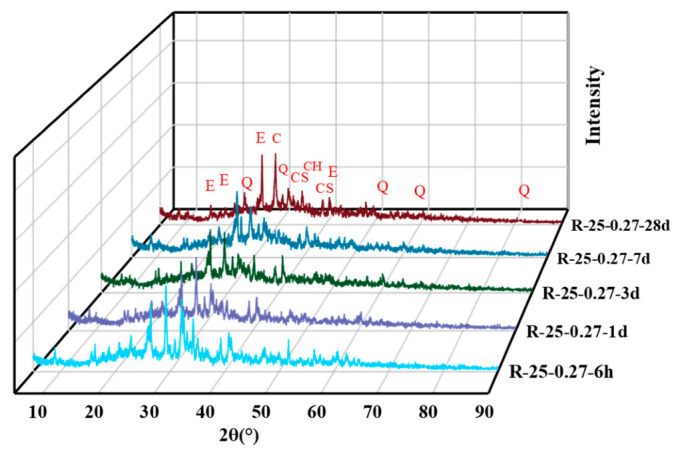
XRD pattern of RHE-ECC under a 25% SAC substitution rate.

The rapid reduction of setting time with increasing SAC content can be explained from the perspective of hydration kinetics in conjunction with the XRD results (Figure 14) (Figure 14 is presented in Section 3.4.1 XRD). XRD analysis indicates that at 6 h of hydration, the C_4_A_3_S^−^ peak (CS) was already clearly identifiable alongside prominent AFt peaks, demonstrating that the hydration reaction had progressed substantially within the initial hours. The rapid formation of needle-like AFt crystals creates a three-dimensional interlocking network between particles, rapidly consuming free water and bridging particles, which is the direct microscopic cause of setting acceleration. The relationship between SAC content and initial setting time is approximately hyperbolic: increasing SAC from 25% to 50% reduced the initial setting time by approximately 60% (from 25 min to ~10 min), whereas further increase from 50% to 100% produced only an additional 40% reduction. This nonlinear relationship reflects competition for sulfate and water among the growing number of ye’elimite grains at higher SAC contents.

Considering the initial setting time, the incorporation of fibers imposes higher demands on the mixing process. A longer mixing time further compresses the available window for casting. Therefore, this study suggests that the initial setting time should be maintained at approximately 25 min to ensure a sufficient casting window.

#### 3.1.2. Compressive Strength and Flexural Strengths

**Figure 5:**
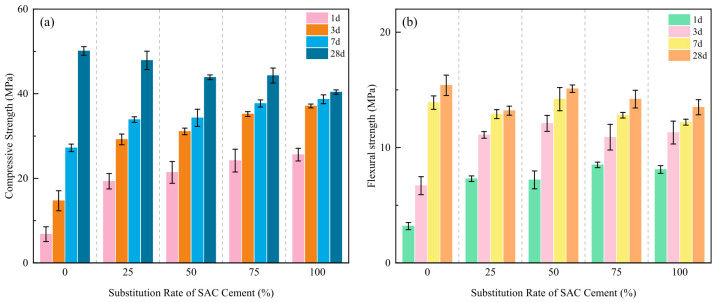
Compressive strength and flexural strength of RHE-ECC prepared with various substitution rates of SAC cement: (**a**) compressive strength; (**b**) flexural strength.

Figure 5a presents the compressive strength development of RHE-ECC with varying SAC replacement levels at different curing ages. The incorporation of SAC significantly alters the compressive strength evolution of RHE-ECC, with all groups exhibiting a characteristic pattern of rapid early-age strength gain followed by a decelerated or even declining growth rate at later ages.

In terms of early-age strength, higher SAC replacement levels correspond to greater 1 d compressive strength. The pure OPC group achieved only 6.8 MPa at 1 d, while the SAC-25 through SAC-100 groups reached 19.3, 21.4, 24.2, and 25.6 MPa, respectively, representing improvements of 183.8–276.5%, attributed to the rapid hydration of C_4_A_3_S^−^ in SAC generating abundant AFt and quickly establishing a load-bearing framework [40,41].

However, the early-age strength gain exhibited diminishing marginal returns: the increment from 0% to 25% was 12.5 MPa, whereas that from 75% to 100% was only 1.4 MPa. Regarding long-term strength, the 28 d compressive strength decreased with increasing SAC replacement level, from 50.1 MPa for the pure OPC group to 47.9, 43.9, 44.3, and 40.4 MPa for SAC-25 through SAC-100, corresponding to reductions of 4.4–19.4%. This is because higher SAC content reduces the OPC proportion, limiting C-S-H gel formation from continued C_3_S hydration and constraining later-age strength development. The 7 d to 28 d strength increment decreased from 22.9 MPa for pure OPC to only 1.7 MPa for SAC-100, further confirming that later-age hydration tends toward stagnation at high SAC dosages. The above hydration mechanism interpretation for the rapid early-age strength development is based on the XRD phase analysis results of this study (substantial AFt formation detected at 6 h, as shown in Figure 14) and hydration kinetics studies reported in the literature, rather than direct calorimetric or porosity measurements from this study. For reference, Winnefeld and Lothenbach [18] reported that pure SAC cement releases approximately 70–80% of its total hydration heat within the first 24 h, compared to only 20–30% for OPC during the same period. García-Maté et al. [12] demonstrated through MIP that SAC-blended systems exhibited a total porosity reduction of 35–45% at 1 d compared to pure OPC systems at the same age, which is consistent with the significantly higher 1 d compressive strength of the SAC groups observed in the present study compared to the pure OPC group.

Figure 5b presents the flexural strength of RHE-ECC at various curing ages under different SAC replacement levels.

**Figure 15:**
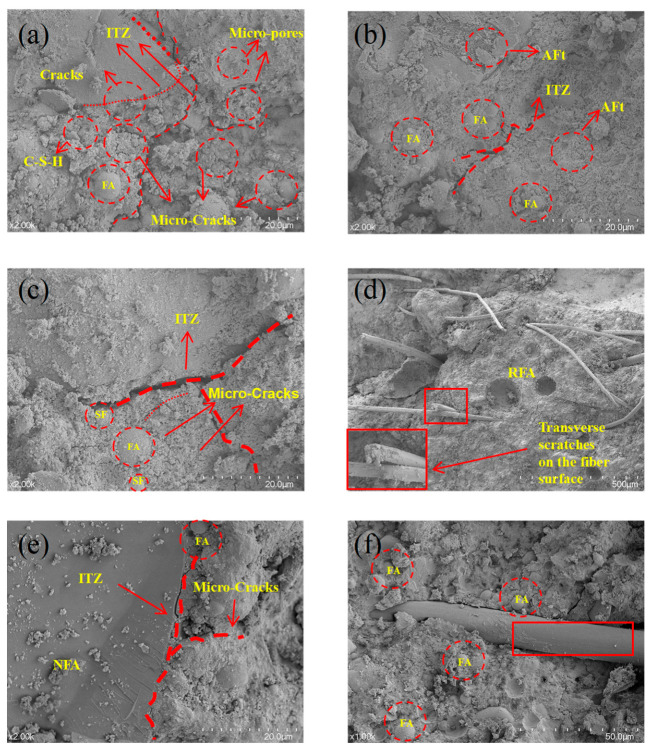
28-d SEM micrographs: (**a**–**d**) microstructural morphology of RFA-ECC; (**e**,**f**) microstructural morphology of NFA-ECC.

The pure OPC group achieved a 1 d flexural strength of only 3.2 MPa, while the SAC-25 through SAC-100 groups reached 7.3, 7.2, 8.5, and 8.1 MPa, respectively, representing improvements of 125–165.6%. By 3 d, all SAC groups exceeded 10.9 MPa, over 1.6 times that of the OPC group. However, unlike compressive strength, the 28 d flexural strength differences among groups were considerably more convergent, all falling within 13.2–15.4 MPa with a maximum reduction of only 14.3%. This phenomenon indicates that the fiber bridging effect exerts a compensatory effect on matrix strength variations under flexural loading mode. This inference is consistent with the morphological features of fibers bridging across crack surfaces observed by SEM in Figure 15d (Figure 15 is presented in Section 3.4.2 SEM). Notably, the SAC-25 group exhibited a slightly lower 28 d flexural strength (13.2 MPa) with an increment of only 0.3 MPa from 7 d to 28 d, possibly associated with the competitive effect between OPC and SAC hydration products within the ITZ at this particular mix proportion. From a hydration mechanism perspective, the essential role of SAC replacement level lies in regulating the rate of early-age AFt framework construction and the quantity of later-age C-S-H gel formation, while simultaneously influencing the extent of the pozzolanic reaction of FA—when SAC replacement is excessively high, insufficient OPC content leads to reduced Ca(OH)_2_ generation, which lowers the degree of FA reaction and consequently constrains later-age strength development.

In summary, the incorporation of SAC markedly enhances early-age compressive strength through the rapid formation of AFt; however, its dilution effect on the OPC component restricts later-age C-S-H gel production, leading to a decline in long-term strength with increasing replacement level. The SAC-25 group achieves a favorable balance between early- and later-age strength by providing rapid early-age strength development while retaining a sufficient OPC content to sustain later-age hydration.

#### 3.1.3. Tensile Performance

**Figure 6:**
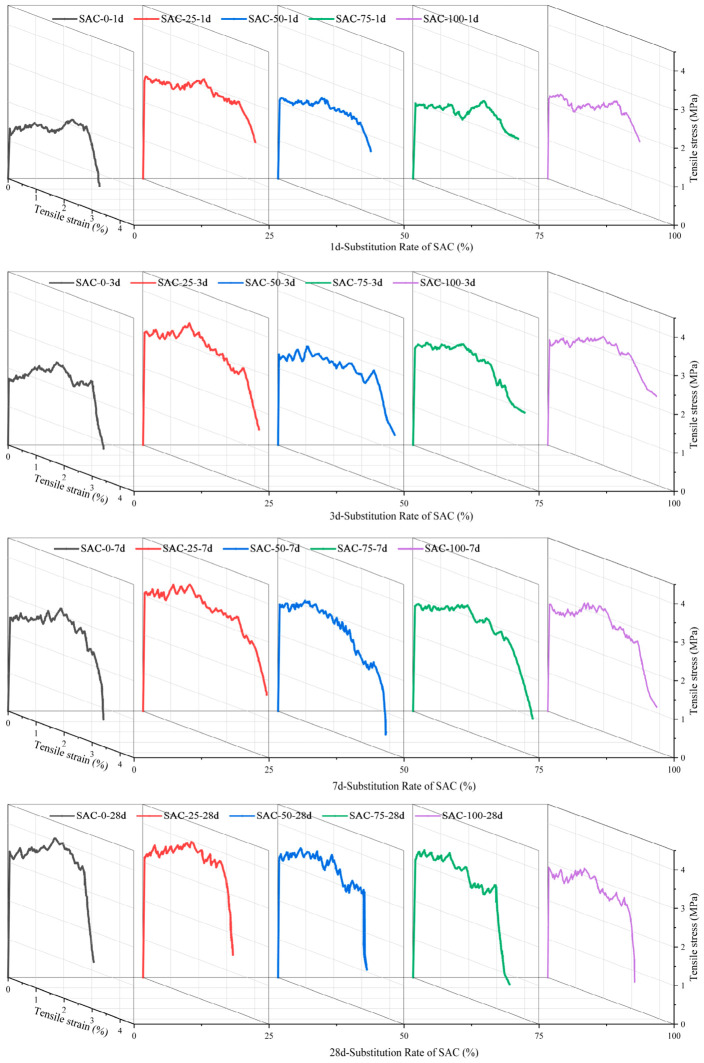
Uniaxial tensile stress-train curves of RHE-ECC at 1, 3, 7, and 28 d under various substitution rates of SAC.

**Figure 7:**
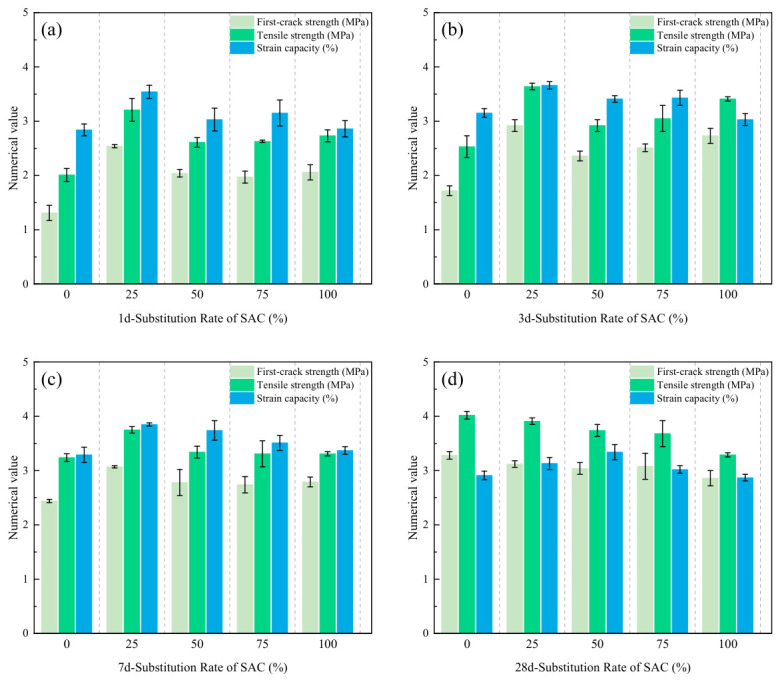
First-crack strength (MPa), tensile strength (MPa), and strain capacity (%) at different curing ages with varying SAC substitution rates. (**a**) 1 d curing; (**b**) 3 d curing; (**c**) 7 d curing; (**d**) 28 d curing.

The stress–strain curves obtained from uniaxial tensile tests on RHE-ECC specimens with different SAC replacement levels are presented in Figure 6, and the tensile mechanical properties of ECC specimens at various curing ages are shown in Figure 7. The initial cracking strength is defined as the stress value corresponding to the onset of the strain-hardening branch, the maximum tensile stress is defined as the peak tensile strength of RHE-ECC, and the ultimate tensile strain is defined as the strain value corresponding to the termination point of the strain-hardening branch.

From the perspective of material design, the first-crack strength (*σ_fc_*) represents the effective design strength of RHE-ECC, as it defines the upper boundary of the elastic working range and corresponds to the onset of cracking. The ultimate tensile strength (*σ_tu_*) serves as additional information indicating the load-carrying reserve beyond the elastic limit.

The strain-hardening behavior is confirmed through the stress–strain curves: all SAC-incorporated specimens exhibited pronounced serrated fluctuations during the strain-hardening stage, with each fluctuation corresponding to the formation of a new crack. The ultimate tensile strains (2.51–3.85%) far exceeded the elastic limit (approximately 0.02%), directly demonstrating the establishment of a saturated multiple-cracking pattern.

With increasing curing age, both the initial cracking strength and ultimate tensile strength of the RHE-ECC specimens exhibited an upward trend. Taking the group with a SAC replacement level of 25% as an example, its 1 d tensile strength reached 3.21 MPa, corresponding to 88.2%, 85.6%, and 82.1% of the 3 d, 7 d, and 28 d tensile strengths, respectively, indicating that RHE-ECC had already undergone a relatively thorough degree of early-age hydration within 1 d. As the curing age further increased, unhydrated reactive particles continued to participate in hydration reactions and the matrix densification progressively improved; the fracture toughness (*K_m_*) is expected to increase accordingly based on established micromechanical theory [42,43]. Although *K_m_* was not directly measured in this study, the increase in compressive strength and initial cracking strength with curing age provides indirect evidence supporting this inference.

Regarding the ultimate tensile strain, all groups of RHE-ECC specimens exhibited a trend of initial increase followed by a subsequent decrease with curing age, generally reaching a peak at the 7 d curing age and then gradually declining thereafter. This is attributed to two concurrent mechanisms: on the one hand, the matrix fracture toughness *K_m_* increases with curing age, which in turn raises the crack tip fracture toughness (*J_tip_*); on the other hand, the continuous evolution of fiber–matrix interfacial properties lead to a reduction in the maximum complementary energy at the fiber bridging site (*J*’*_b_*) (energy criterion). The combined effect of these two factors results in a decline in the pseudo-strain-hardening index Pseudo-Strain-Hardening (*PSH)* (*PSH* = *J*’*_b_*/*J_tip_*), which exerts an adverse influence on the ductility and toughness of RHE-ECC. The *PSH* theory, proposed by Li and Leung [42,43], establishes two necessary conditions for ECC to achieve steady-state multiple cracking: (i) the energy criterion, requiring that the maximum fiber-bridging complementary energy *J*’*_b_* exceed the crack-tip energy *J_tip_* (= *K_m_^2^*/*E_m_*); and (ii) the strength criterion, requiring that the fiber-bridging peak stress σ_0_ exceed the matrix-cracking stress *σ_fc_*. Both conditions must be simultaneously satisfied for the formation of each new crack. It is worth noting that the high tensile strain capacity of RHE-ECC facilitates the release of strain energy that may accumulate during the hardening shrinkage process of concrete, which can, to a certain extent, prevent early-age cracking and thereby contribute to enhancing the fatigue durability and extending the service life of pavement overlays. This reduction in *J*’*_b_* is attributed to the deposition of hydration products on fiber surfaces, as can be verified from Figure 15d (Figure 15 is presented in Section 3.4.2 SEM).

Among all groups with different replacement levels, the group with a SAC replacement level of 25% maintained a relatively high 28 d tensile strength of 3.91 MPa while preserving a favorable ultimate strain of 3.13%. This is because 25% SAC provided an early-age strength framework for the material, while the remaining 75% OPC supplied an adequate yet not excessive amount of Ca(OH)_2_ as an activator for the pozzolanic reaction of FA at later ages. This moderate level of activation ensured the later-age tensile load-bearing capacity of the matrix while avoiding excessive densification of the fiber–matrix interface, thereby circumventing the later-age embrittlement phenomenon observed in the pure OPC group and achieving a favorable synergy between strength and ductility across all curing ages. This replacement level not only compensated for the deficiencies of the pure OPC system, namely insufficient early-age strength and excessive later-age embrittlement, but also effectively avoided the later-age strength deterioration encountered in high SAC replacement level systems (SAC replacement level > 50%), which arises from self-desiccation effects and the inability to activate the pozzolanic reaction of FA due to insufficient Ca(OH)_2_. The RHE-ECC with a SAC replacement level of 25% was capable of providing a tensile strength of 3.21 MPa at 1 d while maintaining an ultimate strain of 3.13% at 28 d. This synergistic performance of high strength and high ductility across all curing ages provides a theoretical basis for the engineering application of this novel RHE-ECC material.

### 3.2. Effect of Water–Binder Ratio

#### 3.2.1. Compressive Strength and Flexural Strength

**Figure 8:**
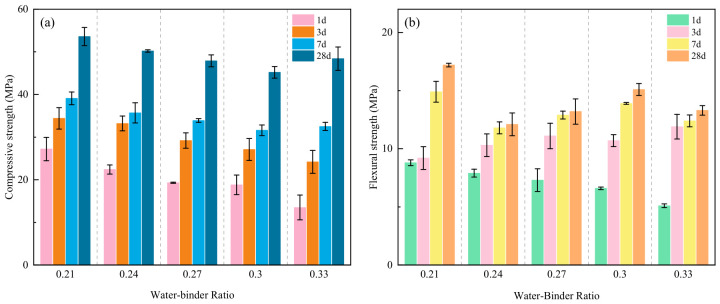
Compressive strength and flexural strength of RHE-ECC prepared with various W/B ratios: (**a**) compressive strength; (**b**) flexural strength.

Figure 8a presents the compressive strength of RHE-ECC at various curing ages under different W/B ratios.

At the 1 d curing age, the compressive strength of RHE-ECC decreased monotonically with increasing W/B ratio, with a reduction of up to 50.37% between the highest and lowest groups. This is because, under compressive loading, the influence of the W/B ratio on matrix densification predominates over its effect on fiber–matrix interfacial properties. As the W/B ratio increases, the packing density of the paste decreases and the initial porosity increases, thereby resulting in a reduction in compressive strength. At the 1 d and 3 d curing ages, a clear negative correlation was observed between compressive strength and W/B ratio, indicating that the initial packing density of the paste plays a dominant role in governing the mechanical performance at this stage. At a W/B ratio of 0.21, the proportion of free water is low, and the AFt generated by SAC hydration interweaves and consolidates within the limited pore space, forming a dense early-age skeletal structure, with 1 d and 3 d compressive strengths reaching 27.2 MPa and 34.4 MPa, respectively. As the W/B ratio increased to 0.33, the excess mixing water occupied the space that would otherwise have been filled by hydration products, weakening the distribution density and connectivity of the AFt framework, and the 1 d compressive strength of the 0.33 group dropped to 13.5 MPa. As the curing age progressed to 28 d, the matrix densification process gradually transitioned from early-age AFt framework construction to the deep hydration of OPC components. The R-25-0.21 (RFA, 25% SAC, W/B = 0.21) group maintained the highest value of 53.6 MPa at 28 d. Within the 0.24–0.30 range, the 28 d compressive strength continued to follow a decreasing trend, declining from 50.2 MPa to 45.2 MPa. The 28 d compressive strength of the R-25-0.33 group (48.4 MPa) was slightly higher than those of the R-25-0.27 group (47.9 MPa) and the R-25-0.30 group (45.2 MPa); however, this group exhibited greater data scatter than the other groups. Considering the influence of experimental variability, the differences in 28 d strength among these three groups are relatively small and should not be over-interpreted mechanistically. Overall, the general trend of decreasing 28 d compressive strength with increasing W/B ratio remains valid.

Figure 8b presents the flexural strength of RHE-ECC at various curing ages under different W/B ratios. At 1 d, flexural strength decreased monotonically with increasing W/B ratio, with the five groups from R-25-0.21 to R-25-0.33 (RFA, 25% SAC, W/B = 0.33) measuring 8.81, 7.9, 7.3, 6.6, and 5.1 MPa, respectively. At this stage, hydration was in its early phase and matrix strength dominated the contribution to flexural performance; although a lower W/B ratio reduced paste fluidity and increased the difficulty of eliminating internal defects, this was insufficient to offset the positive effect of matrix strength. The period from 1 d to 3 d represented the fastest stage of flexural strength growth, with an average growth rate of approximately 14.61% across all groups. As hydration progressed, the advantages of better workability and fewer internal defects in higher W/B ratio groups gradually emerged, and the negative correlation between W/B ratio and flexural strength weakened. By 7 d, hydration of the SAC component had approached completion, the strength-growth rate decelerated, and inter-group differences were no longer significant. The 28 d values were approximately comparable to those at 7 d, indicating that flexural strength had essentially stabilized after 7 d. The influence of W/B ratio on flexural strength was most pronounced at early ages; with increasing curing age, continuous filling by hydration products progressively bridged the initial defects in higher W/B ratio groups, and inter-group differences gradually converged.

#### 3.2.2. Tensile Performance

**Figure 9:**
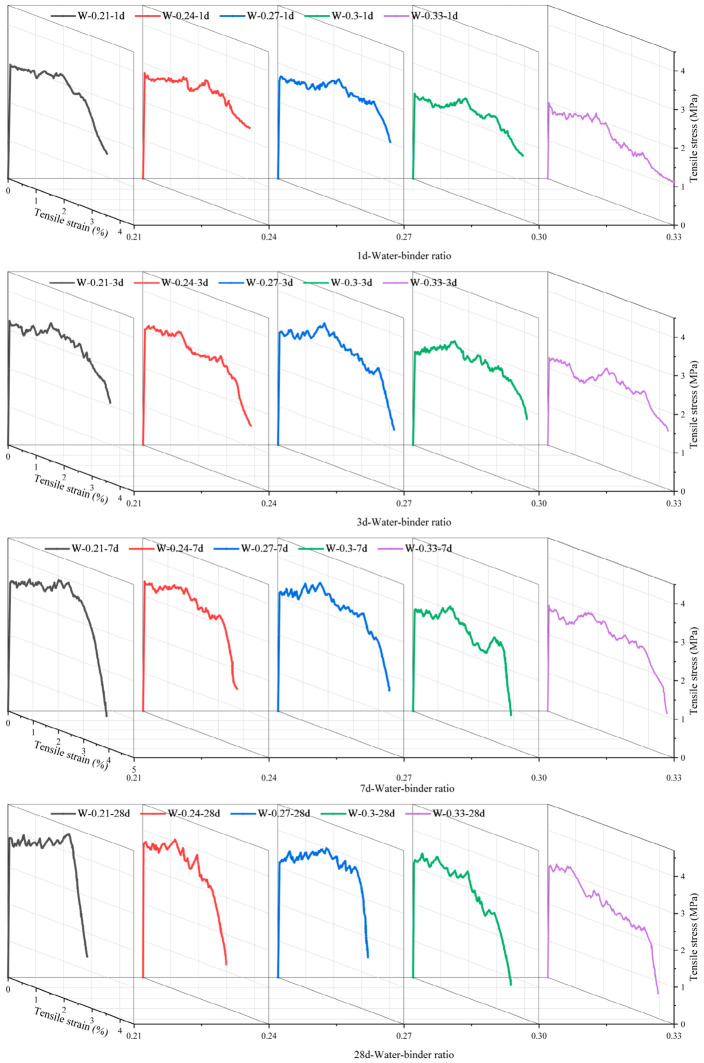
Uniaxial tensile stress–strain curves of RHE-ECC at 1, 3, 7, and 28 d under various W/B ratios.

**Figure 10:**
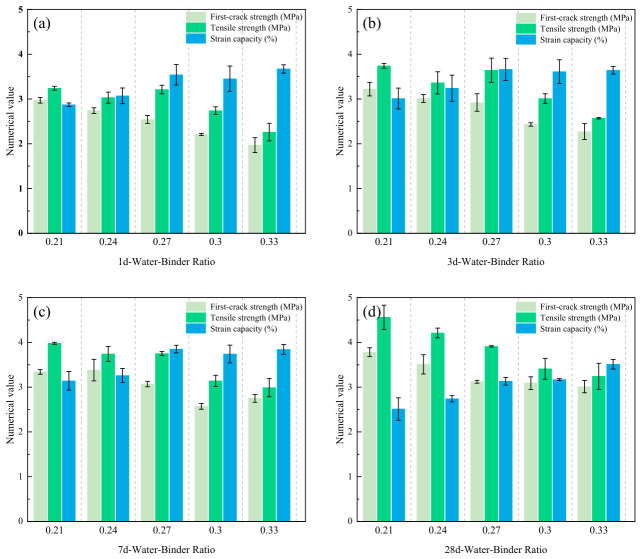
First-crack strength (MPa), tensile strength (MPa), and strain capacity (%) at different curing ages with varying W/B ratios. (**a**) 1 d curing; (**b**) 3 d curing; (**c**) 7 d curing; (**d**) 28 d curing.

The W/B ratio is a critical parameter governing the matrix densification and the fiber–matrix interfacial mechanical response of ECC, and its essence lies in balancing the competitive relationship between strength and strain-hardening capacity. The stress–strain curves obtained from uniaxial tensile tests on RHE-ECC specimens with a 25% SAC replacement level at different W/B ratios are presented in Figure 9, and the tensile mechanical properties of ECC specimens at various curing ages are shown in Figure 10.

The experimental results indicated that the influence of the W/B ratio on the mechanical properties of RHE-ECC exhibited a consistent pattern across different curing ages: strength decreased monotonically with increasing W/B ratio, while ductility showed an increasing trend. At the 1 d curing age, since the pozzolanic activity of FA had not yet been activated, the matrix strength relied primarily on the early-age hydration products of SAC and OPC. As the W/B ratio increased from 0.21 to 0.33, the initial cracking strength decreased from 2.97 MPa to 1.97 MPa, representing a reduction of 33.7%, while the ultimate strain increased from 2.87% to 3.67%. This inverse variation indicates that under high W/B ratio conditions, the matrix is relatively porous, allowing fibers to undergo progressive frictional slippage at lower stress levels, which is conducive to the development of early-age deformation capacity. As the curing age increased, the degree of hydration continued to improve and the strength of all groups continued to develop; however, the stratified pattern of strength differentiation among different W/B ratios persisted throughout. The 7 d curing age represented a critical stage for ductility development, with the 0.27 group achieving the highest ultimate strain of 3.85% across all curing ages, suggesting that an ideal balance between matrix fracture toughness and fiber bridging complementary energy was attained at this stage. By the 28 d curing age, the continued densification of the matrix compressed the available space for fiber slippage, and the ductility of all groups generally declined. The ultimate strain of the low W/B ratio group (0.21) dropped to 2.51%, exhibiting a significant loss in ductility. The high W/B ratio groups (0.30 and 0.33), owing to the retention of a certain level of porosity within the matrix, still maintained relatively high ductility values of 3.17% and 3.51%, respectively; however, their ultimate tensile strengths were only 3.41 MPa and 3.24 MPa, with the strain-hardening capacity approaching its lower limit [44].

The elastic strain limit (*ε_e_*), defined as the strain corresponding to the first-crack point (the termination of the linear elastic range governed by Hooke’s law), was determined from the uniaxial tensile stress–strain curves. For the recommended mix design R-25-0.27, the elastic strain limit at 28 d was approximately 0.017–0.021%, with a corresponding first-crack stress of 3.12 MPa and an elastic modulus of E = *σ_fc_*/*ε_e_* ≈ 18.2 GPa. Beyond this point, the material transitions into the strain-hardening regime, where deformation is accommodated through progressive multiple cracking rather than elastic deformation.

Based on a comprehensive analysis, reducing the W/B ratio enhances matrix strength, but excessively high interfacial bond forces tend to induce brittle fiber rupture and suppress multiple cracking. Conversely, increasing the W/B ratio favors steady-state fiber pull-out and multiple crack propagation, albeit at the expense of strength.

The 0.27 group achieved an initial cracking strength of 3.12 MPa, an ultimate tensile strength of 3.91 MPa, and an ultimate strain of 3.13% at the 28 d curing age, with all three indicators positioned at an upper-intermediate level. At the fiber–matrix interface level, a higher W/B ratio produces a more porous ITZ around the fibers, reducing *τ*_0_ and *G_d_*. According to the micromechanical model of Leung and Li [42], a moderate reduction in *τ*_0_ shifts the fiber failure mode from rupture-dominated toward pull-out-dominated, increasing the effective fiber pull-out length and enhancing *J*’*_b_*. The W/B ratio of 0.27 represents the optimal condition where *J_tip_* is sufficiently reduced while *J*’*_b_* remains adequately high, yielding the maximum *PSH* index and consequently the best ultimate tensile strain. This group established a rational balance among matrix densification, interfacial bond strength, and fiber slippage space, and is therefore identified as the optimal W/B ratio for achieving the best overall tensile performance in this system.

### 3.3. Comparison of Mechanical Properties Between RFA and NFA

#### 3.3.1. Compressive Strength and Flexural Strengths

**Figure 11:**
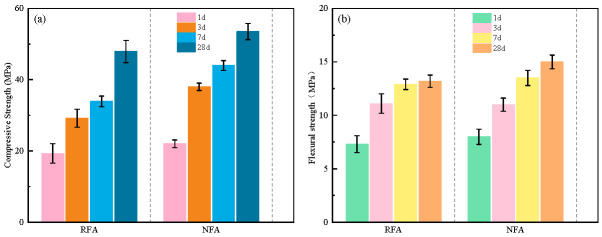
Compressive and flexural strengths of RHE-ECC with RFA and NFA at various ages: (**a**) compressive strength; (**b**) flexural strength.

Figure 11 presents the compressive and flexural strength development of RFA-ECC and NFA-ECC at a SAC replacement level of 25%.

In terms of compressive strength comparison, the NFA group exhibited higher values than the RFA group at all curing ages. At the 1 d curing age, the difference between the two groups was relatively small, with the NFA group achieving approximately 22 MPa and the RFA group approximately 19.5 MPa. This is because the rapid early-age hydration of SAC dominates the strength development at this stage, and the influence of aggregate type on early-age strength has not yet fully manifested. From the 3 d to 7 d curing ages, the gap between the two groups widened considerably, with the NFA group reaching approximately 38 MPa and 44 MPa, respectively, while the RFA group attained only approximately 29.5 MPa and 33.5 MPa. This is primarily attributable to the high porosity and low intrinsic strength of the old mortar layer adhering to the surface of recycled sand, which results in a relatively weak ITZ between the recycled aggregate and the new paste, constituting the principal limiting factor for mid-age strength development. By the 28 d curing age, the gap between the two groups narrowed to some extent [27]. The RFA group exhibited a strength gain of approximately 14 MPa from 7 d to 28 d, representing a substantial increase, which indicates that the C-S-H gel continuously generated by later-age OPC hydration progressively filled the interfacial defects surrounding the recycled sand, partially compensating for the mid-age strength deficit. The 28 d compressive strengths of the NFA and RFA groups were approximately 53 Mpa and 47.5 Mpa, respectively, corresponding to a reduction of approximately 10.4%, which remains within an engineering-acceptable range.

Regarding flexural strength, the differences between the two groups at all curing ages were considerably smaller than those observed in compressive strength. At the 1 d curing age, the flexural strengths of the two groups were approximately comparable. From the 3 d to 28 d curing ages, the values were concentrated within the range of approximately 10 Mpa–12.5 Mpa, with the inter-group differences further diminishing. This indicates that the bridging and toughening effect of PVA fibers played a dominant role in flexural performance, effectively compensating for the matrix strength loss and interfacial defects induced by the recycled sand. Furthermore, the flexural strength growth of both groups tended toward stagnation during the 7 d to 28 d stage, further corroborating the observation that the flexural strength of this system had essentially stabilized after 7 d.

#### 3.3.2. Tensile Performance

**Figure 12:**
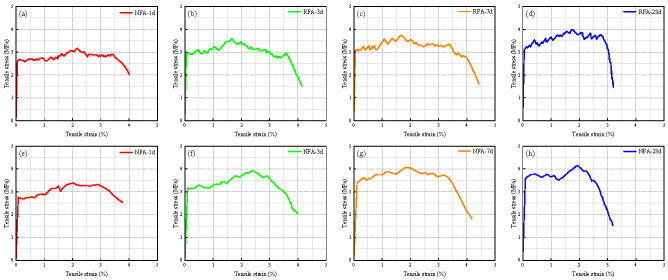
Tensile stress-strain curves of RHE-ECC at different curing ages for RFA and NFA groups. (**a**) RFA-1d; (**b**) RFA-3d; (**c**) RFA-7d; (**d**) RFA-28d; (**e**) NFA-1d; (**f**) NFA-3d; (**g**) NFA-7d; (**h**) NFA-28d.

**Figure 13:**
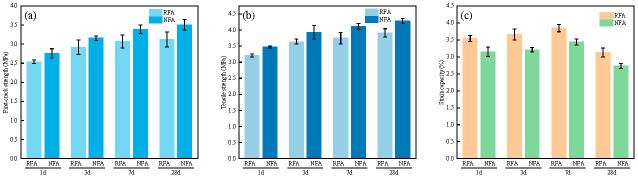
Tensile mechanical properties of RHE-ECC with RFA and NFA at different curing ages. (**a**) First-crack strength; (**b**) Tensile strength; (**c**) Strain capacity.

The stress–strain curves obtained from uniaxial tensile tests on RFA-ECC and NFA-ECC specimens at a SAC replacement level of 25% are presented in Figure 12, and the tensile mechanical properties of ECC specimens at various curing ages are shown in Figure 13.

At all curing ages, the initial cracking strength and ultimate tensile strength of the NFA specimens were higher than those of the RFA specimens, and both exhibited an increasing trend with curing age. The initial cracking strength essentially reflects the cracking resistance of the matrix, which is jointly controlled by the defect sensitivity and the fracture toughness of the matrix. NFA particles are dense and free from adhered old mortar, forming a more compact ITZ with hydration products and exhibiting lower porosity. Li et al. [26] reported through BSE-SEM image analysis that the ITZ porosity in RFA concrete (48–55% within 0–20 μm from aggregate surface) was approximately 1.8–2.2 times that of NFA concrete (25–30%) for aggregates with comparable water absorption. Xiao et al. [34] demonstrated through nanoindentation that the average micro-hardness within the ITZ of RFA was 30–40% lower than that of NFA, with the mechanically weak zone extending to approximately 40–55 μm from the aggregate boundary. As the hydration process progressed, the filling effect of C-S-H gel and pozzolanic reaction products on the ITZ in the NFA system was more pronounced, causing the defect disparity between the two matrices to further widen at later ages. This constitutes the primary reason for the progressively increasing difference in initial cracking strength with curing age. The ultimate tensile strength is governed by the peak load-carrying capacity of the fiber bridging stress, in which the anchoring efficiency of the matrix on the fibers is a key factor. The denser matrix in the NFA system provided stronger anchoring constraints on the fibers, enabling them to transmit higher bridging stresses during the debonding–slippage process at the crack face, thereby enhancing the ultimate tensile strength. This observation is consistent with the experimental results reported by Li and Cheng et al. [45,46]. It is noteworthy that the increment in ultimate tensile strength (8.1–9.6%) was slightly lower than the increment in initial cracking strength at later ages (10.4–12.5%), indicating that the ratio between the two (i.e., the strain-hardening margin) was narrower in the NFA system compared to the RFA system. This observation provides a mechanistic precursor for explaining the subsequent differences in ultimate strain. The ultimate tensile strain of the RFA group was superior to that of the NFA group. This phenomenon can be systematically explained from the perspective of the *PSH* micromechanical criterion [42,43]. The necessary conditions for ECC to achieve steady-state multiple cracking are that the crack-tip complementary energy *J_tip_* must be less than the fiber-bridging complementary energy *J*’*_b_*, and that the initial cracking strength σ*_fc_* must be less than the ultimate tensile strength *σ_tu_* (strength criterion). An increase in the matrix fracture toughness *K_m_* directly elevates *J_tip_*, compresses the energy margin, reduces the number of saturated cracks, and consequently lowers the ultimate strain. Due to the porous old mortar layer and weak ITZ, RFA objectively reduces the matrix *K_m_* value, enabling cracks to initiate more readily at lower stress levels and propagate in a steady-state manner, thereby forming a denser multiple cracking pattern that macroscopically manifests as a higher ultimate strain [47,48]. The porous old mortar layer inherent to RFA reduces the matrix fracture toughness, enabling cracks to initiate sequentially at lower stress levels and propagate in a steady-state manner, thereby forming a denser saturated multiple-cracking pattern. This regulatory effect can be quantitatively characterized through the fluctuation features of the stress–strain curves: the serrated oscillations in the strain-hardening stage directly reflect the number of cracks, with each ‘rise–drop’ cycle corresponding to the formation of a new crack. Comparison of the 28 d stress–strain curves in Figure 12 reveals that RFA-ECC exhibits notably more fluctuation cycles than NFA-ECC, with smaller drop amplitudes per cycle, indicating a greater number of cracks and more effective fiber bridging in the RFA system. Macroscopically, the ultimate tensile strain of RFA-ECC (3.13%) is superior to that of NFA-ECC (2.71%), and the *σ_tu_*/*σ_fc_* ratio is higher, confirming the effective regulation of multiple cracking behavior by RFA.

In contrast, the excessively dense matrix of the NFA system leads to an elevated *K_m_*, which suppresses the multiple cracking capability.

Based on the comprehensive analysis above, from the perspective of material design, the application of RFA in RHE-ECC not only achieves the environmental benefit of solid-waste resource utilization but also leverages its microstructural characteristics to regulate the matrix fracture toughness. With only a moderate reduction in strength (8–12%), the strain-hardening capacity and ductility performance of the material can be enhanced, which is of critical importance for the crack resistance and energy dissipation functions of ECC. The aforementioned results provide a theoretical basis from the micromechanical perspective for the rational application of recycled aggregates in high-ductility cementitious composites.

### 3.4. Micromorphology Analysis

#### 3.4.1. XRD

XRD patterns were collected using a Rigaku SmartLab diffractometer (Rigaku, Tokyo, Japan) with Cu-Kα radiation (λ = 1.5406 Å), operating at 40 kV and 40 mA. Scans were performed over a 2θ range of 5–90° with a step size of 0.02° and a scanning rate of 2°/min. Prior to XRD analysis, hydration was arrested by the solvent exchange method: crushed sample fragments were immersed in isopropanol for 7 d, followed by vacuum drying at 40 °C for 48 h to effectively terminate hydration reactions and minimize carbonation.

Figure 14 presents the XRD patterns of RHE-ECC with a SAC replacement level of 25% at different curing ages (6 h, 1 d, 3 d, 7 d, and 28 d) (E-Aft, C-C_2_S, Q-SiO_2_, CS-C_4_A_3_S^−^, CH-Ca(OH)_2_).

At 6 h of hydration, the quartz (Q) peak was relatively prominent, which is associated with the substantial amount of SiO_2_ present in the 40% FA incorporated in the system. The AFt and CS peaks were also clearly identifiable, indicating that C_4_A_3_S^−^ rapidly hydrated upon contact with water to generate AFt and AH_3_, reflecting the rapid setting and hardening characteristics of SAC. The formation of ettringite crystals and their associated expansive effect exert a significant influence on the mechanical properties of SAC cement-based matrices [49,50]. At this stage, no CH peak was detected, suggesting that OPC hydration was still in the induction period. SF, owing to its high pozzolanic activity, may consume trace amounts of CH at early ages, accelerating cement hydration and shortening the induction period, thereby rendering the CH peak undetectable.

From 1 d to 3 d of hydration, the AFt peak intensified significantly while the CS peak tended to disappear, indicating the continued progression of C_4_A_3_S^−^ hydration. The CH peak appeared at 1 d and reached a relatively high intensity by 3 d, suggesting that C_3_S hydration had entered the acceleration period, generating C-S-H gel and CH. During this stage, the Q peak exhibited minimal change, indicating that the pozzolanic reaction of FA had not yet been initiated on a large scale.

At 7 d of hydration, the CH peak was notably weaker compared to that at 3 d, indicating that the pozzolanic reaction had been clearly activated. SF, owing to its large specific surface area and high reactivity, preferentially consumed CH to generate secondary C-S-H. Simultaneously, the SiO_2_ and Al_2_O_3_ in FA gradually dissolved under the high-pH environment, producing secondary C-S-H and C-A-H. The synergistic action of these two pozzolanic materials accelerated the consumption of CH.

At 28 d of hydration, the CH peak was further significantly weakened. Since the OPC content accounted for only 37.5% of the cementitious system, the total amount of CH generated was limited, while the 50% pozzolanic materials continuously consumed CH, reducing the residual quantity to a relatively low level. The AFt peak remained stable, and the CS peak had almost entirely disappeared. Within the 2θ range of 25–35°, the baseline exhibited a noticeable elevation, indicating a significant increase in amorphous phase content, primarily comprising AH_3_ alumina gel and C-S-H gel generated by pozzolanic reactions.

In summary, the system with a 25% SAC replacement level exhibited multi-stage synergistic hydration characteristics: SAC rapidly generated AFt within 6 h, imparting early strength; OPC underwent accelerated hydration from 1 d to 3 d, producing C-S-H and CH; SF initiated secondary hydration around 7 d, synergistically consuming CH together with FA and generating secondary C-S-H and C-A-H gels. These hydration products effectively filled the matrix pores and optimized the pore structure, thereby providing assurance for the sustained development of later-age mechanical properties and long-term durability.

#### 3.4.2. SEM

SEM observations were performed using a TESCAN MIRA LMS field-emission scanning electron microscope at an accelerating voltage of 10–15 kV and a working distance of 8–12 mm, employing the secondary electron (SE) imaging mode. Samples were obtained from fracture surfaces after tensile testing, mounted on aluminum stubs with conductive carbon tape, and sputter-coated with a gold layer of approximately 10 nm thickness to ensure surface conductivity. All subfigures of Figure 15 were captured at a magnification of 2000× and 1000×.

To further elucidate the mechanisms by which RFA and NFA influence the microstructure of the ECC matrix and the fiber–matrix interfacial characteristics, SEM observations were conducted on the fracture surfaces of both groups of specimens, and the results are presented in Figure 15.

Figure 15a displays the morphology of the old mortar layer adhering to the surface of RFA particles. It can be clearly observed that the old mortar layer exhibits a loose structure with a large number of irregularly distributed micro-pores and a small amount of residual unhydrated cement particles. This characteristic indicates that the old mortar layer of RFA underwent varying degrees of damage and deterioration during the original concrete service and the crushing process, resulting in a porosity significantly higher than that of the natural aggregate matrix, with a distinctly heterogeneous distribution of hydration products. This porous old mortar layer constitutes the primary source of defects within the RFA-ECC matrix and makes a direct contribution to the reduction of *K_m_*. This is similar to the findings reported by Ding et al. [51].

Figure 15b further presents the microstructural morphology of the ITZ at the junction between the old and new mortar in the RFA system. Within this region, the oriented stacking of layered hydration products (CH and low-density C-S-H gel) and micro-cracks distributed along the interface can be observed. The formation of this phenomenon is attributable to the high-water absorption rate of the old mortar layer of RFA. During the initial mixing stage, the old mortar layer preferentially absorbs the surrounding free water, causing a localized increase in the effective water-to-cement ratio at the interface, which in turn promotes the formation of a highly oriented CH crystal enrichment zone and a loose C-S-H gel network in this region. Consequently, the RFA system effectively possesses a dual ITZ structure (i.e., the old mortar–original aggregate interface and the new paste–old mortar interface), in which the new-to-old mortar ITZ becomes the mechanically weakest region within the entire matrix due to the aforementioned moisture migration effect.

Figure 15c illustrates the crack propagation path within the RFA-ECC matrix. It can be observed that a through-going crack preferentially propagated along the porous regions of the old mortar and the weak zones of the new-to-old mortar ITZ, with the crack path exhibiting a pronounced tortuous deflection pattern. This phenomenon confirms the crack-path deflection mechanism in the RFA system: the old mortar layer and its surrounding ITZ, serving as pre-existing microstructural defects within the matrix, provide preferential sites for crack initiation while simultaneously guiding the crack tip to propagate in a steady-state manner along low-toughness paths [52,53]. From a micromechanical perspective, these defects effectively reduce the equivalent fracture toughness *K_m_* of the matrix, maintaining the energy release rate at the crack tip at a relatively low level, thereby broadening the stress range over which the *PSH* energy criterion (*J*’*_b_*/*J_tip_* > 1) is satisfied and creating favorable mechanical conditions for the formation of multiple cracking.

Figure 15d presents the fiber-bridging morphology in RFA-ECC. Multiple PVA fibers were observed bridging across the crack face at various inclination angles to achieve effective bridging, with varying degrees of matrix debris adhering to the fiber surfaces. Simultaneously, spherical FA particles and circular holes left by fiber pull-out were visible on the crack face. The matrix adherents on the fiber surfaces and the intact fiber morphology indicate that the fibers underwent a complete debonding–frictional slippage–pull-out process during crack opening, rather than experiencing brittle rupture. This pull-out-dominated failure mode implies that individual fibers continuously dissipated energy throughout the entire pull-out stroke, allowing the fiber bridging complementary energy (*J*’*_b_*) to be fully mobilized and providing the requisite energy assurance for the strain-hardening behavior of ECC.

Figure 15e presents the matrix morphology and fiber–matrix interfacial characteristics of NFA-ECC. In stark contrast to the RFA system, the matrix structure of the NFA system was notably denser and more homogeneous, with hydration products exhibiting a continuous distribution and a significant reduction in both the number and size of micro-pores and micro-cracks. The fine spherical particles dispersed throughout the matrix were identified as unreacted FA and SF particles, indicating that the mineral admixtures still possessed sustained potential pozzolanic reactivity.

Figure 15f presents the typical morphology of fiber pull-out in NFA-ECC. It can be observed that the surfaces of the pulled-out fibers were relatively smooth, and the matrix surrounding the fibers remained intact with no visible interfacial damage zone. These characteristics indicate that the chemical bond strength between the fibers and the matrix in the NFA system was excessively high, requiring the fibers to overcome substantial interfacial shear stress during the debonding stage, which led to a shortened effective pull-out length and insufficient energy dissipation during the frictional slippage stage. Compared with the abundant matrix adherents on the fiber surfaces and the multi-fiber synergistic bridging features observed in the RFA system in Figure 15d, the excessively strong interfacial bond in the NFA system restricted, to a certain extent, the full mobilization of the fiber-bridging complementary energy *J*’*_b_*, which was unfavorable for the full development of a saturated multiple-cracking pattern.

### 3.5. Mechanism of Multiple Cracking Induced by RFA

According to the study by Kirthika and Singh [25], RFA with water absorption in the range of 7–10% typically corresponds to a residual mortar content of 30–45% and an internal porosity of 25–35%. The high-water absorption of the RFA used in this study (8.71%) indirectly confirms the presence of a substantial porous old mortar phase.

The serrated fluctuations during the strain-hardening stage of the stress–strain curves (Figure 12) can serve as an indirect quantitative indicator of multiple-cracking behavior: each ‘rise–drop’ cycle corresponds to the formation of a new crack, and the number of fluctuations reflects the crack count. Comparing the 28 d stress–strain curves, RFA-ECC exhibited notably more fluctuations than NFA-ECC, with smaller individual drop amplitudes, indicating a greater number of cracks and more effective fiber bridging in the RFA system. Furthermore, the higher ultimate tensile strain and *σ_tu_*/*σ_fc_* ratio of RFA-ECC corroborate its superior multiple cracking capability from a macroscopic perspective. Based on the combined evidence of tensile performance data (higher ultimate strain and strain-hardening margin), stress–strain curve fluctuation characteristics (more fluctuation cycles and smaller drop amplitudes), and qualitative SEM morphological observations, it can be reasonably inferred that the porous old mortar layer and weakened ITZ of RFA are conducive to regulating the multiple-cracking mechanism.

Although direct measurement of *PSH* parameters was not conducted in this study, an order-of-magnitude estimation can be provided based on values from the literature for similar PVA-ECC systems. For PVA fibers in cementitious matrices with a compressive strength of 40–55 MPa, representative interfacial parameters reported in the literature include the following: chemical bond strength *G_d_* = 1.0–2.5 J/m^2^, frictional bond stress *τ*_0_ = 1.5–3.0 MPa, and slip-hardening coefficient *β* = 0.3–0.8 [41,47]. For the NFA-ECC matrix (28 d compressive strength ≈ 53 MPa): estimated *K_m_* ≈ 0.35–0.45 MPa·m^1/2^, *E_m_* ≈ 20 GPa, yielding *J_tip_* ≈ *K_m_*^2^/*E_m_* ≈ 6.1–10.1 J/m^2^. For the RFA-ECC matrix (28 d compressive strength ≈ 47.9 MPa): accounting for the porosity-induced *K_m_* reduction of approximately 15–20%, estimated *K_m_* ≈ 0.28–0.38 MPa·m^1/2^, yielding *J_tip_* ≈ 3.9–7.2 J/m^2^. With *J*’*_b_* typically in the range of 10–20 J/m^2^ for PVA-ECC systems [47,48], the estimated *PSH* energy index (*J*’*_b_*/*J_tip_*) is approximately 1.0–3.3 for NFA-ECC and approximately 1.4–5.1 for RFA-ECC. The higher *PSH* index of RFA-ECC is consistent with its experimentally observed superior strain-hardening capacity.

**Figure 16:**
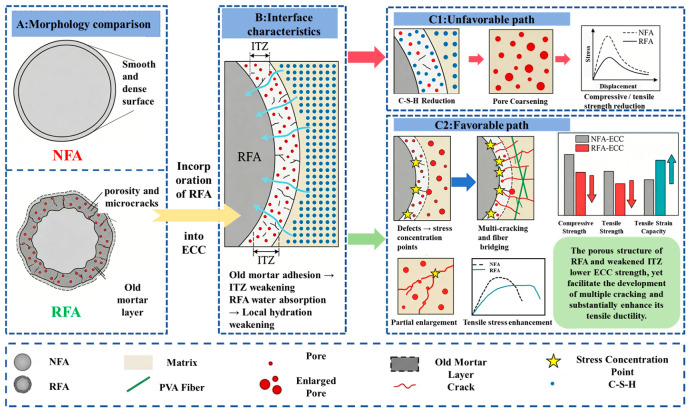
Schematic of multiple-cracking behavior of RHE-ECC.

It can thus be concluded that the substitution of natural sand with recycled sand exerted a certain adverse effect on the compressive strength of RHE-ECC, primarily stemming from the lower intrinsic strength of recycled sand and its weak ITZ; however, the reduction at 28 d was only 10.4%. The influence on flexural strength was negligible, as the bridging and toughening effect of PVA fibers effectively compensated for the matrix defects, demonstrating favorable applicability of recycled sand ECC in terms of flexural performance. The detailed mechanistic diagram is presented in Figure 16. Note that the morphological characteristics of NFA and RFA depicted in this schematic are inferred from measured physical properties (Table 3) and supported by petrographic observations reported in the literature for similar RFA [25,26,34], rather than direct petrographic analysis of the specific materials used in this study. The RFA shown in sections B and C represents multi-particle assemblages rather than individual particles. Actual particle size range is as follows: 0.075–1.18 mm; fiber length: 12 mm >> max. aggregate particle size: 1.18 mm. Old mortar layer: ~20–200 μm (estimated from Refs. [26,51]); new-to-old mortar ITZ: ~30–55 μm (estimated from Ref. [34]); NFA-paste ITZ: ~20–30 μm (estimated from Ref. [34]). Illustrations are not to scale.

SEM observations revealed that the NFA surface was relatively smooth and dense, with no apparent pores or cracks, whereas the RFA surface was rougher and more irregular, exhibiting a greater number of pores and micro-cracks. This is attributed to the residual old mortar from the original concrete adhering to the RFA surface, which sustained damage and defects during the crushing process.

The proposed mechanistic interpretation is supported by evidence from three complementary levels: (i) at the matrix defect level—Figure 15a,c demonstrates the porous structure of the old mortar and the tortuous crack deflection path along the weakened ITZ regions, corresponding to a reduction in *K_m_*; (ii) at the fiber–matrix interface level—Figure 15d (RFA-ECC: matrix debris adhering to fiber surfaces, indicating a complete debonding–pull-out process) contrasts with Figure 15f (NFA-ECC: smooth fiber surfaces with intact surrounding matrix, indicating excessively strong interfacial bond), reflecting differences in energy dissipation efficiency; (iii) at the macroscopic performance level—the ultimate tensile strain of RFA-ECC was superior to that of NFA-ECC at all curing ages (Figure 13c), validating the combined effect of the above mechanisms.

Although petrographic thin-section analysis was not performed in this study, the fundamental differences between RFA and NFA are quantitatively reflected in their measured physical properties (Table 3). The water absorption of RFA (8.71%) is approximately 5.1 times that of NFA (1.71%), and the apparent density of RFA (2560 kg/m^3^) is 2.2% lower than that of NFA (2617 kg/m^3^). According to the study by Kirthika and Singh [25], RFA with water absorption in the range of 7–10% typically corresponds to a residual mortar content of 30–45% and an internal porosity 3–5 times higher than that of natural sand. The significantly elevated water absorption of the RFA used in this study provides quantitative physical evidence confirming the presence of a substantial porous old mortar phase. For petrographic reference, Li et al. [26] conducted thin-section analysis on RFA of similar origin (normal-strength concrete, C30–C40 grade) and particle size range (0.075–1.18 mm), and reported that RFA particles comprised approximately 35–50% residual mortar by area, characterized by a porous and heterogeneous microstructure. Given that the RFA in the present study was produced through a similar crushing and sieving process, it is reasonable to infer comparable petrographic characteristics.

The pores and micro-cracks on the RFA surface exert multifaceted influences on the mechanical properties of ECC materials. On the one hand, the ITZ between RFA and the fresh cementitious paste exhibits relatively weaker bond performance due to the presence of the old mortar. The old mortar layer thickness and ITZ width were not directly measured in this study. Based on quantitative characterization results from the literature for RFA with comparable physical properties, (i) Xiao et al. [34] reported through nanoindentation and BSE-SEM that the ITZ width between recycled fine aggregate and new paste was approximately 30–55 μm, approximately 1.5–2.0 times that of the NFA system (20–30 μm); (ii) for fine-fraction RFA with particle size < 1.18 mm, the old mortar layer thickness is geometrically constrained by the particle size and typically ranges from 20–200 μm [26,51]. These literature values are adopted as reference parameters in the mechanistic discussion and are explicitly identified as estimates rather than measured values. Concurrently, RFA possesses a higher water absorption capacity, absorbing free water from the matrix during the hydration process, which leads to a reduced degree of hydration in the ITZ region, a corresponding decrease in the amount of C-S-H hydration products generated, and an increase in internal capillary pore pressure, thereby enlarging the pore structure of the ECC matrix. These factors act in combination to reduce, to a certain extent, the compressive strength and tensile strength of ECC materials incorporating RFA [52].

The pore defects and weaker ITZs introduced by RFA constitute multiple stress-concentrating weak points within the matrix, causing cracks to propagate preferentially along weakened ITZs or larger pores, thereby promoting multiple-cracking behavior. During this process, PVA fibers distribute tensile strain among multiple fine cracks through their bridging action, significantly enhancing the tensile strain capacity of ECC. Therefore, in the SAC–OPC composite cementitious system, although the porous structure of RFA has a certain adverse effect on strength, its defect characteristics facilitate the establishment of a multiple-cracking mechanism, enabling the material to exhibit superior tensile ductility.

## 4. Conclusions

(1) The OPC–SAC composite binder with 25% SAC replacement level achieves an optimal balance between rapid early-age strength and long-term mechanical performance. The RHE-ECC attains a 1 d compressive strength of 19.3 MPa (183.8% higher than the pure OPC group) while maintaining a 28 d compressive strength of 47.9 MPa, with an initial setting time of approximately 25 min that ensures adequate casting workability for fiber-reinforced composites.

(2) At a water–binder ratio of 0.27, RHE-ECC achieves the best comprehensive tensile performance at 28 d, with a first-crack strength of 3.12 MPa, an ultimate tensile strength of 3.91 MPa, and an ultimate tensile strain of 3.13%, representing a rational balance among matrix densification, interfacial bond strength, and fiber slippage capacity.

(3) Within the SAC–OPC composite system, the full substitution of NFA with RFA reduces the 28 d compressive strength by only 10.4%, while enhancing the ultimate tensile strain by approximately 15% due to the porous old mortar layer and weakened ITZ, which lower the matrix fracture toughness and promote multiple cracking, demonstrating favorable engineering applicability for green rapid-repair materials.

It should be noted that the matrix fracture toughness *K_m_* was not directly measured in this study; future work will quantitatively characterize it through notched-beam bending tests or wedge splitting tests to validate the PSH energy criterion. The residual mortar content of RFA was not systematically quantified; future work will combine the acid dissolution method, MIP, and nanoindentation to establish quantitative relationships between residual mortar parameters and ECC ductility. This study did not involve elastic modulus or durability evaluation; future work will systematically assess elastic modulus, interface bond strength, deformation compatibility, and durability (freeze–thaw resistance, chloride penetration resistance, carbonation resistance, etc.).

## Figures and Tables

**Table 1:** Chemical compositions of cement, FA, and SF.

Chemical Composition (%)	SAC	OPC	FA	SF
CaO	43.69	67.63	4.43	1.33
SiO_2_	6.25	25.72	52.27	97.02
Al_2_O_3_	22.14	3.16	31.56	0.68
SO_3_	13.91	3.50	1.47	0.25
Fe_2_O_3_	1.31	3.50	5.58	0.16

**Table 2:** Properties of PVA fibers.

Length (mm)	Diameter (mm)	Young’s Modulus (GPa)	Density (g/cm^3^)	Tensile Strength (MPa)	Elongation (%)
12	0.015	40	1.28	1830	7

**Table 3:** Physical properties of aggregates.

Aggregate Type	Apparent Density (kg/m^3^)	Water Absorption (%)	Fineness Modulus
NFA	2617	1.71	2.15
RFA	2560	8.71	1.81

**Table 4:** Mixture proportion of ECC (kg/m^3^).

Mixture ID	SAC	OPC	FA	SF	Effective Water	Absorbed Water	RFA	NFA	Fiber	HRWR	Total
N-25-0.27	161.6	484.8	517.1	129.3	349.1	8.0	0	465.4	25.80	10.3	2151.4
R-0-0.27	0	644.0	515.2	128.8	347.8	40.4	463.7	0	25.80	10.3	2176.0
R-25-0.27	161.0	483.0	515.2	128.8	347.8	40.4	463.7	0	25.80	10.3	2176.1
R-50-0.27	322.0	322.0	515.2	128.8	347.8	40.4	463.7	0	25.80	10.3	2176.0
R-75-0.27	483.0	161.0	515.2	128.8	347.8	40.4	463.7	0	25.80	10.3	2176.0
R-100-0.27	644.0	0	515.2	128.8	347.8	40.4	463.7	0	25.80	10.3	2176.0
R-25-0.21	174.6	523.9	558.8	139.7	293.4	43.8	503.0	0	25.80	11.2	2274.2
R-25-0.24	167.5	502.6	536.1	134.0	321.7	42.0	482.5	0	25.80	10.7	2222.9
R-25-0.30	155.0	464.9	495.9	124.0	371.9	38.9	446.3	0	25.80	9.9	2132.6
R-25-0.33	149.4	448.1	478.0	119.5	394.3	37.5	430.2	0	25.80	9.6	2092.4

Note: Each mix proportion was named following the format fine aggregate type—Substitution rate of SAC cement—W/B with N representing NFA and R representing RFA.

## Data Availability

The original contributions presented in this study are included in the article. Further inquiries can be directed to the corresponding author.
